# Prospective case-control analysis of the aetiologies of acute undifferentiated fever in Vietnam

**DOI:** 10.1080/22221751.2019.1580539

**Published:** 2019-03-04

**Authors:** Nhiem Le-Viet, Viet-Nho Le, Hai Chung, Duc-Tuan Phan, Quang-Duong Phan, Thanh-Van Cao, Cédric Abat, Didier Raoult, Philippe Parola

**Affiliations:** a Aix Marseille University, IRD, AP-HM, SSA, VITROME, Marseille, France; b IHU-Méditerranée Infection, Marseille, France; c Department of Tropical Medicine, Quang Nam Central General Hospital, Quang Nam, Vietnam; d School of Medicine and Pharmacy, Danang University, Danang, Vietnam; e Department of Internal Medicine II, Quang Nam Northern Mountainous Region General Hospital, Quang Nam, Vietnam; f Department of Internal Medicine B, Quang Nam Regional General Hospital, Quang Nam, Vietnam; g Department of Tropical Medicine, Quang Nam Provincial General Hospital, Quang Nam, Vietnam; h Aix Marseille University, IRD, AP-HM, MEPHI, Marseille, France

**Keywords:** Acute undifferentiated fever, aetiologies, rickettsial infections, *Leptospira*, dengue, influenza

## Abstract

Acute undifferentiated fever (AUF) is frequently observed in tropical settings, but diagnosing the cause of AUF is often a challenge for local physicians and the physicians treating returning travellers. We conducted a case-control study in central Vietnam in 2016. A total of 378 febrile adult patients (AUFs) with a fever for ≤21 days, no evidence of localized infection and negative screening tests for dengue and malaria, and 384 afebrile adult patients (Controls) were prospectively enrolled. Whole blood, plasma, eschar swab, throat swab and urine specimens were collected and analysed. Quantitative PCR and RT-PCR were used to test for 55 bacteria, viruses and their subtypes. Serological tests were also used to test for rickettsial agents. The most common aetiology was influenza virus (20.9% in AUFs vs. 0% in Controls), followed by rickettsial agents (mainly *Orientia tsutsugamushi* and *Rickettsia typhi*) (10.8% vs. 0.3%), dengue virus (7.7% vs. 0.5%), *Leptospira* (4.8% vs. 0.8%), adenovirus (4.8% vs. 1.0%), and enterovirus (2.1% vs. 0%) (*p* < .05). The real proportion of dengue in AUF cases was underestimated because patients with dengue-positive rapid diagnosis tests were excluded from the study. The emerging agent *Rickettsia felis*, which had not been previously observed in Vietnam, was detected in this study. In total, 216 patients (57.1%) were given causative diagnoses, comprising 143 (66.2%) monoinfections and 73 (33.8%) coinfections. The infections caused by these agents should be considered in clinical practice and further studies. Additionally, agents susceptible to doxycycline were detected in 15.6% of AUFs; thus, this drug should be included in the panel used to treat AUF patients.

## Introduction

Acute undifferentiated fever (AUF) is a temporary febrile illness accompanied by nonspecific manifestations [1]. It is frequently observed in clinical practice, but the diagnosis of its causes, particularly in developing and tropical countries, is often a challenge for clinicians due to the diversity of potential causes and the limited availability of diagnostic tools [1,2]. In addition, the research on febrile illness has many gaps, such as the limited epidemiological knowledge of fevers, the use of nonspecific tests and the lack of control groups, which limit the ability to definitively conclude the role of an infectious agent as the cause of AUF [1,3]. Therefore, prospective studies enrolling patients with AUF and controls, using paired sera for serological tests as well as direct methods of pathogen detection, are necessary to better decipher the aetiologies of AUF. Accordingly, the results obtained from such studies can lead to recommendations regarding prophylactic and/or treatment measures for local populations and travellers.

Vietnam has been highly successful in controlling malaria, but despite the rapid decline in malaria, fever caused by nonmalarial aetiologies remains a common reason for hospital admission [4]. Although AUF is observed daily in hospitals, the precise cause is usually not known due to the lack of diagnostic tools in most hospitals in the country. In a few recent studies, dengue infection (33.6%), influenza (4.1%) and other respiratory viral infections (5.3%) were reported as common causes of AUF in southern Vietnam [5,6]. However, these studies likely focused only on some pathogen groups of viruses or bacteria and did not include afebrile controls. In a case-control study with 1193 febrile patients and 282 afebrile individuals conducted in Cambodia, which shares a border with Vietnam, the most frequent pathogens detected were *Plasmodium vivax* (33.4%), *Plasmodium falciparum* (26.5%), *Leptospira* (9.4%), *influenza virus* (8.9%), Dengue virus (6.3%) and rickettsial agents (4.1%), but a significant proportion of malaria parasites and *Leptospira* were also observed in the control group [7]. Such data require careful interpretation of the role of pathogens in febrile illness, and more clinical studies are needed to verify the findings.

We thus conducted a prospective study using reference methods and appropriate control group that aimed to detect the aetiologies of fever in patients with AUF in Vietnam.

## Results

### Participant characteristics

**Figure 1.**
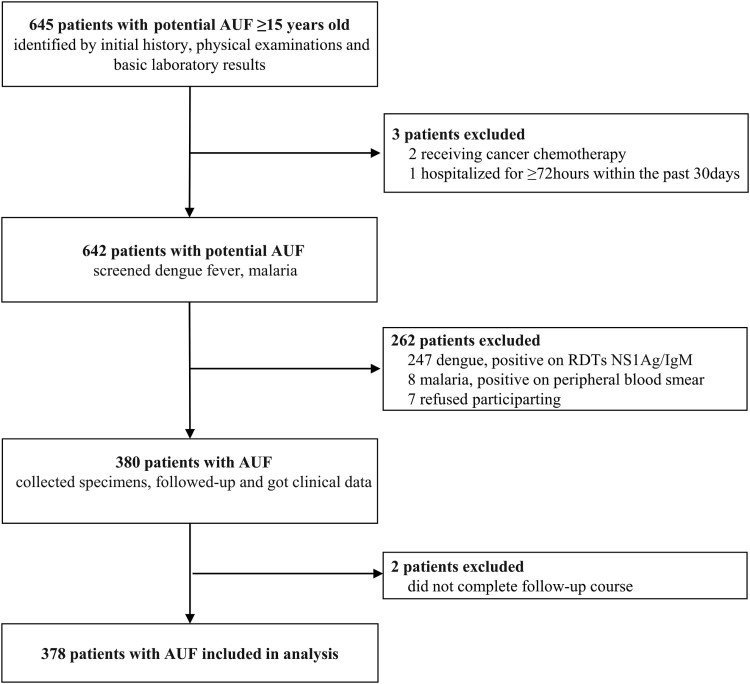
Flowchart of the enrolment of patients with AUF.

Between May and August 2016, a total of 762 adult participants, including 378 (43.4% male) AUF participants (AUFs) and 384 (43.0% male) afebrile participants (Controls), were included in this analysis (Figure 1). The median (interquartile range [IQR]) ages of the AUFs and Controls were 38 (25–53) and 42 (27–56.5) years, respectively.

Of the 378 AUFs, 168 patients (44.4%) were farmers, 41 patients (11.1%) performed forest-related activities, and 38 patients (10.1%) had a chronic disease or comorbid condition (Table 1). Patients with AUF usually had high fever and a short course of illness, with a mean peak temperature of 39.2°C (95% CI: 39.1–39.3) and a median febrile duration of 5 (4–6) days. The most common accompanying symptom was headache (81.0%), followed by disappetite (54.5%), muscle pain (44.3%), dizziness (37.5%), cough (30.4%), back pain (28%) and other less frequent symptoms. A small proportion of AUFs also presented some clinical signs, such as rash (4.3%), lymphadenopathy (4.0%), eschar (3.2%), haemorrhage (2.4%), hepatomegaly (0.8%) and splenomegaly (0.5%). Shock, altered mental status, and seizures were not observed in our patients. The overall median (IQR) white blood cell count (WBC) was 6.9 (5–9.1) × 10^9^/L, and approximately one-fifth of the patients presented with an abnormal WBC classified as either leukocytosis (WBC > 12 × 10^9^/L; 35 [9.3%] patients) or leukopenia (WBC < 4 × 10^9^/L; 50 [13.2%] patients). The overall median (IQR) platelet count (PLT) was 176.5 (133–223) × 10^9^/L, and one-third of the patients (129 patients; 34.1%) exhibited thrombocytopenia (PLT < 150 × 10^9^/L). The median (IQR) values of the aspartate aminotransferase level (AST) and alanine aminotransferase level (ALT) were 31.5 (23–61.5) and 26.5 (15.5–49.5) IU/L, respectively. Abnormal aminotransferase levels were observed in 116 (30.7%) patients, which presented either an elevated level of one (elevated AST in 21 cases, elevated ALT in 6 cases) or both of the enzymes (89 cases). Abdominal ultrasound confirmed some cases presenting with hepatomegaly or splenomegaly but did not indicate other abnormal signs. The chest X-ray and urine analysis results did not indicate specific abnormalities in any cases. No patients were excluded because of being human immunodeficiency virus (HIV) positive. No specific aetiological diagnoses were made for any febrile patients during the sampling period.

**Table 1.** Clinical characteristics of the patients with AUF (*n* = 378).

**Characteristics**	**Frequency**	**Percentage**
Forest exposure^a^	41	11.1
Comorbid conditions		
Alcohol abuse	1	0.3
Basedow	1	0.3
Chronic renal failure	2	0.5
Diabetes	1	0.3
Essential thrombocytopenia	1	0.3
Gout	1	0.3
Hypertension	27	7.1
Pregnancy	4	1.1
Headache	306	81.0
Dizziness^b^	141	37.5
Disappetite^b^	205	54.5
Nausea^c^	68	18.1
Vomiting^c^	34	9.1
Diarrhoea^c^	46	12.3
Sore throat	65	17.2
Breathlessness^c^	4	1.1
Cough	115	30.4
Chest pain^c^	7	1.9
Abdominal pain^c^	50	13.3
Back pain^c^	105	28
Muscle pain^c^	166	44.3
Joint pain^d^	59	15.8
Rash^c^	16	4.3
Haemorrhage^d^	9	2.4
Eschar	12	3.2
Hepatomegaly^⁑^	3	0.8
Splenomegaly^⁑^	2	0.5
Lymphadenopathy^e^	15	4.0
** **	**Mean**	**Standard deviation**
Peak body temperature (°C)	39.2	0.8
	**Median**	**Interquartile range**
Fever duration (days)	5	4–6
White blood cell count (k/µL)	6.9	5–9.1
Neutrophil (k/µL)	4.8	2.9–6.4
Lymphocyte (k/µL)	1.3	0.8–1.9
Monocyte (k/µL)	0.6	0.4–0.9
Hemoglobin (g/dL)	13	12–14
Platelet count (k/µL)	176.5	133–223
Aspartate aminotransferase (IU/L)^f^	31.5	23–61.5
Alanine aminotransferase (IU/L)^f^	26.5	15.5–49.5

Notes: ^⁑^Clinicians assessed the patients clinically and confirmed by ultrasound.

^a^Data are missing in 7 cases; ^b^data are missing in 2 cases; ^c^data are missing in 3 cases; ^d^data are missing in 4 cases; ^e^data are missing 1 case; ^f^data are missing in 86 cases.

Whole blood, plasma, urine and throat swab specimens were collected from all the participants at the time of enrolment in the study. In addition, 260 (68.8%) convalescent-phase plasmas were obtained from patients with AUF who returned to the hospital after discharge. Additionally, eschar swab specimens were collected for aetiology analyses from 10 of 12 AUF patients presenting an eschar.

### Infectious disease agents identified

#### Vector-borne diseases agents and Leptospira

Rickettsial agents, dengue virus and *Leptospira* were identified in 41 (10.8%), 29 (7.7%) and 18 (4.8%) AUFs, respectively, and in one (0.3%), two (0.5%) and three (0.8%) Controls, respectively (*p* < .05) (Table 2). Among the rickettsial agents detected in AUFs, *O. tsutsugamushi* was found in more than half of the cases (21/41 patients; 51.2%), and *R. typhi* (18/41 patients; 43.9%) and *R. felis* (2/41 patients; 4.9%) were found in smaller proportions of cases (Table S1). *R. felis* was also detected in one control. *Anaplasma* spp., *Bartonella* spp., *Borrelia* spp., or *C. burnetii* were not found. Among the dengue agents observed in AUFs, dengue virus serotype-1 (DEN-1) was found in slightly more than three-quarters of the patients (22/29 patients; 75.9%), and DEN-4 (5 patients; 17.2%) and DEN-2 (2 patients; 6.9%) were detected in smaller proportions of the patients.

**Table 2.** Aetiologies detected in whole blood, plasma and urine.

Aetiologies	AUFs (*n* = 378)	Controls (*n* = 384)	*p*-value
***Leptospira*** (qPCR)*, n* (%)	18 (4.8)	3 (0.8)	<.001
***Rickettsia****, n* (%)	41 (10.8)	1 (0.3)	<.001
*O. tsutsugamushi, n* (%)	21 (5.5)	0	
qPCR + IFA (n)	8^a^	–	
qPCR only (n)	11^a^	0	
IFA only (n)	2	–	
*R. typhi, n* (%)	18 (4.8)	0	
qPCR + IFA (n)	4	–	
qPCR only (n)	6	0	
IFA + WB (n)	1	–	
IFA only (n)	7	–	
*R. felis, n* (%)	2 (0.5)	1 (0.3)	
qPCR + IFA (n)	0	–	
qPCR only (n)	2^b^	1	
IFA only (n)	0	–	
**Dengue virus** (qRT-PCR)*, n* (%)	29 (7.7)	2 (0.5)	<.001
DEN-1*, n* (%)	22 (5.8)	0	
DEN-2*, n* (%)	2 (0.5)	0	
DEN-4*, n* (%)	5 (1.3)	2 (0.5)	

Notes: qPCR = real-time polymerase chain reaction, qRT-PCR = real-time reverse-transcription polymerase chain reaction, IFA = indirect immunofluorescence assay, WB = western blot.

^a^Four cases were positive in both eschar and whole blood specimens.

^b^One case was positive in only the blood specimen, and one case was positive in only the eschar specimen.

#### Respiratory viruses and bacteria

**Figure 2.**
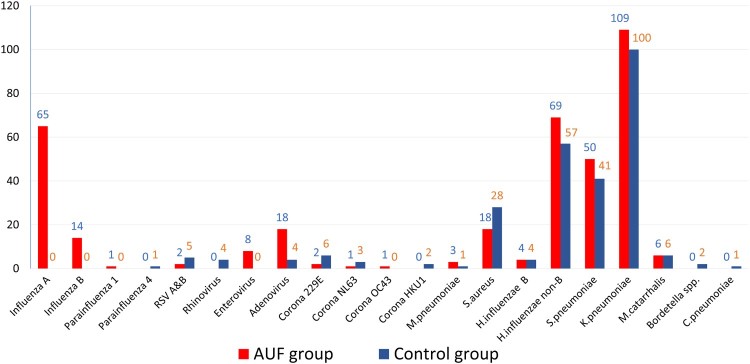
Detected pathogens and their frequency in throat swabs from patients with AUF and controls in central Vietnam.

At least one pathogen was detected in the throat swabs from 246 AUFs (65.1%) and 195 Controls (50.8%) (*p* < .001). In most positive cases (59.4% of AUFs and 71.3% of Controls), a single pathogen was detected, whereas several pathogens were detected in the remaining positive cases (Figure S1). Specifically, six viruses and seven bacteria were found in the throat swabs from the AUFs, whereas five viruses and eight bacteria were found in those from the Controls (Table 3, Figure 2).

**Table 3.** Aetiologies detected in throat swab specimens.

Aetiologies	AUFs (*n* = 378)	Controls (*n* = 384)	*p*-value
Influenza			
Influenza A*, n* (*%*)	65 (17.2)	0	<.001
Influenza B*, n* (%)	14 (3.7)	0	<.001
Parainfluenza 1*, n* (%)	1 (0.3)	0	.32
Parainfluenza 4*, n* (%)	0	1 (0.3)	.32
Enterovirus*, n* (%)	8 (2.1)	0	.004
Adenovirus*, n* (%)	18 (4.8)	4 (1.0)	.002
Rhinovirus*, n* (%)	0	4 (1.0)	.05
RSV A/B*, n* (%)	2 (0.5)	5 (1.3)	.27
Coronavirus			
CoV 229E*, n* (%)	2 (0.5)	6 (1.6)	.16
CoV NL63*, n* (%)	1 (0.3)	3 (0.8)	.32
CoV OC43*, n* (%)	1 (0.3)	0	.32
CoV HKU1*, n* (%)	0	2 (0.5)	.16
*M. pneumoniae, n* (%)	3 (0.8)	1 (0.3)	.31
*S. aureus, n* (%)	18 (4.8)	28 (7.3)	.14
*H. influenzae*			
*H. influenzae* B*, n* (%)	4 (1.1)	4 (1.0)	.98
*H. influenzae* non-type B*, n* (%)	69 (18.3)	57 (14.8)	.21
*S. pneumoniae, n* (%) * *	50 (13.2)	41 (10.7)	.28
*K. pneumoniae, n* (%)	109 (28.8)	100 (26.0)	.39
*M. catarrhalis, n* (%)	6 (1.6)	6 (1.6)	.97
*Bordetella* spp.*, n* (%)	0	2 (0.5)	.16
*C. pneumoniae, n* (%)	0	1 (0.3)	.32

Influenza virus was the most frequently detected virus (79 patients; 20.9%) in AUFs and was not found in Controls (*p* < .001). Similarly, enterovirus was detected in eight (2.1%) patients with AUFs but not in Controls (*p* < .001), and adenovirus was found in 18 (4.8%) patients with AUFs and four (1.0%) Controls (*p* < .05). In contrast, other viruses, including parainfluenza virus, respiratory syncytial viruses (RSV) A/B and coronaviruses, were detected in AUFs and Controls at similar frequencies.

*Klebsiella pneumoniae* was the most common bacterium found in both groups (109 [28.8%] AUFs and 100 [26.0%] Controls). *Haemophilus influenzae* (73 [19.3%] AUFs and 61 [15.9%] Controls) and *Streptococcus pneumoniae* (50 [13.2%] AUFs and 41 [10.7%] Controls) were also commonly detected. Some less common bacteria, including *Staphylococcus aureus, Mycoplasma pneumoniae* and *Moraxella catarrhalis*, were also found in a small number of AUFs and Controls. No significant difference in the proportion of bacteria detected in throat swabs was found between AUFs and Controls.

We also investigated the relationship between the occurrence of viruses and bacteria in AUFs, but the correlation test showed no correlation among the detected viruses and bacteria (Table S2).

#### Multiple pathogen detection

A substantial proportion of pathogen codetection was identified in this study. *O. tsutsugamushi* was present as the single pathogen in 19 patients and was accompanied by *Leptospira* in two patients. Influenza virus was the most frequently observed in codetection cases (46/79; 58%) with one additional pathogen (*K. pneumoniae* [*n* = 15], *H. influenza* [*n* = 6], *S. pneumoniae* [*n* = 5], *Leptospira* [*n* = 3], *S. aureus* [*n* = 1]) or two additional pathogens (*H. influenza* and *K. pneumoniae* [*n* = 6], *H. influenza* and *S. pneumoniae* [*n* = 5], *S. pneumoniae* and *K. pneumoniae* [*n* = 2], coronavirus OC43 and *K. pneumoniae* [*n* = 1], *S. aureus* and *H. influenza* [*n* = 1], *S. aureus* and *K. pneumoniae* [*n* = 1]). Adenovirus (*n* = 9), enterovirus (*n* = 2) and coronavirus 229E (*n* = 1) were also observed in combination with other viruses or bacteria. Other codetections included the detection of two or three bacteria in throat swabs, and *S. pneumoniae*, *K. pneumoniae* and *H. influenza* were the most frequently observed bacteria in these cases.

### Clinical characteristics of the most frequent causes of AUF

Analyses of the clinical characteristics of patients who presented with single scrub typhus, murine typhus, leptospirosis, dengue fever, influenza and other respiratory viral infections are described in Table 4. Patients with scrub typhus were more likely to perform forest-related activities (47.4%) and to have eschar (47.4%) and elevated aminotransaminase (AST, ALT) levels than those with other diagnoses (*p* < .05). Patients with murine typhus had febrile durations (8.5 [6–10] days) longer than those of patients with other diagnoses (except those with scrub typhus), and patients with leptospirosis presented a medium course of fever (5.5 [4–7] days). Patients with dengue fever exhibited lower WBC and PLT counts than nearly all the patients with other diagnoses (*p* < .05). Cough was more frequently observed in patients with influenza (63.6%) than in patients with other diagnoses (*p* < .05). Patients with noninfluenza respiratory viral infection were young, and cough (21.4%) was observed less frequently in these patients than in patients with influenza.

**Table 4.** Clinical characteristics of scrub typhus, murine typhus, leptospirosis, dengue fever, influenza and other respiratory viral infections.

Characteristics	Scrub typhus (*n* = 19)	Murine typhus (*n* = 18)	Leptospirosis (*n* = 12)	Dengue fever (*n* = 28)	Influenza (*n* = 33)	Nonflu RVI (*n* = 14)
Age (years)^a^	47.4 (18.4) ϵ γ	42.7 (17.3) λ	35.4 (18.3)	35.3 (16.1) γ	43.6 (21.3) ‡	28.6 (16.1) ϵ λ ‡
Forest exposure, *n* (%)	9 (47.4) α β γ δ ϵ	0 α	1 (8.3) β	3 (11.1)^c^γ	0 δ	1 (7.1) ϵ
Fever duration (days)^b^	7.5 (5–9.5) δ	8.5 (6–10) ζ η θ λ	5.5 (4–7) ζ	6 (5–7) η σ	5 (4–6) δ θ σ	5 (4–6) λ
Peak T°(°C)^a^	39.2 (0.7)	39.8 (0.8) θ	39.5 (1.1)	39.6 (0.7) σ	39.0 (0.7) θ σ	39.2 (0.8)
Headache, *n* (%)	18 (94.8) β γ	17 (94.4) ζ η	8 (66.7) β ζ	18 (64.3) γ η	26 (78.8)	11 (78.6)
Nausea, *n* (%)	1 (5.3)	3 (16.7)	3 (25)	6 (21.4)	9 (27.3)	4 (28.6)
Vomiting, *n* (%)	1 (5.3)	3 (16.7)	1 (8.3)	2 (7.1)	3 (9.1)	2 (14.3)
Diarrhoea, *n* (%)	0	2 (11.1)	2 (16.7)	3 (10.7)	5 (15.2)	1 (7.1)
Sore throat, *n* (%)	1 (5.3)	2 (11.1)	3 (25)	4 (14.3)	9 (27.3)	4 (28.6)
Cough, *n* (%)	6 (31.6) δ	5 (27.8) θ	1 (8.3) π	3 (10.7) σ	21 (63.6) δ θ π σ ‡	3 (21.4) ‡
Abdominal pain, *n* (%)	5 (26.3)	5 (27.8)	2 (16.7)	5 (17.9)	8 (24.2)	3 (21.4)
Muscle pain, *n* (%)	11 (57.9)	8 (44.4)	6 (50)	10 (35.7)	11 (33.3)	7 (50)
Joint pain, *n* (%)	7 (36.8) γ δ ϵ	5 (27.8) λ	2 (16.7)	4 (14.3) γ	4 (12.1) δ	0 ϵ λ
Rash, *n* (%)	1 (5.3)	3 (16.7) η θ	1 (8.3)	0 η	0 θ	0
Haemorrhage, *n* (%)	0	0	0	2 (7.4)^c^	1 (3.0)	0
Eschar, *n* (%)	9 (47.4) α β γ δ ϵ	0 α	0 β	0 γ	0 δ	0 ϵ
Lymphadenopathy, *n* (%)	8 (42.1) γ δ ϵ	1 (5.6)	1 (8.3)	1 (3.6) γ	0 δ	0 ϵ
WBC (k/µL)^b^	8.2 (5.5–9.1) γ δ	6.3 (5.0–7.9) η	7.4 (6.8–8.5) μ π	4.3 (3.0–6.2) γ η μ φ	5.4 (3.8–7) δ π ‡	8.0 (5.7–9.8) φ ‡
PLT (k/µL)^b^	140.5 (103.5–204)	133.5 (116.5–187) η λ	165.5 (134.5–200.5) μ	118 (65.5–147.5)η μ σ φ	165 (132–189) σ	199.5 (153–228) λ φ
AST (IU/L)^b^	125 (67–157) α β γ δ ϵ	73.5 (34–109) α θ λ	44 (23–66) β	38 (26–74) γ σ	25 (21–29) δ θ σ	39 (16–47) ϵ λ
ALT (IU/L)^b^	106 (77–141) α β γ δ ϵ	50 (27–121) α θ λ	44 (18–53) β	29 (22–45) γ σ	15 (13–27) δ θ σ	26 (13–37) ϵ λ

Notes: Nonflu RVI = noninfluenza respiratory viral infection.

α, β, γ, δ, ϵ, ζ, η, θ, λ, μ, π, ς, σ, φ, ‡ significant difference (*p* < .05) in proportions (chi-square or Fisher’s exact test) or means/medians (Kruskal–Wallis test):

α Scrub typhus vs. Non-ST RI, β Scrub typhus vs. Leptospirosis, γ Scrub typhus vs. Dengue fever, δ Scrub typhus vs. Influenza, ϵ Scrub typhus vs. Noninfluenza RVI, ζ Murine typhus vs. Leptospirosis, η Murine typhus vs. Dengue fever, θ Murine typhus vs. Influenza, λ Murine typhus vs. Noninfluenza RVI, μ Leptospirosis vs. Dengue fever, π Leptospirosis vs. Influenza, ς Leptospirosis vs. Noninfluenza RVI, σ Dengue fever vs. Influenza, φ Dengue fever vs. Noninfluenza RVI, and ‡ Influenza vs. Noninfluenza RVI.

^a^Mean (standard deviation), ^b^median (interquartile range).

^c^Data are missing in 1 case.

**Figure 3.**
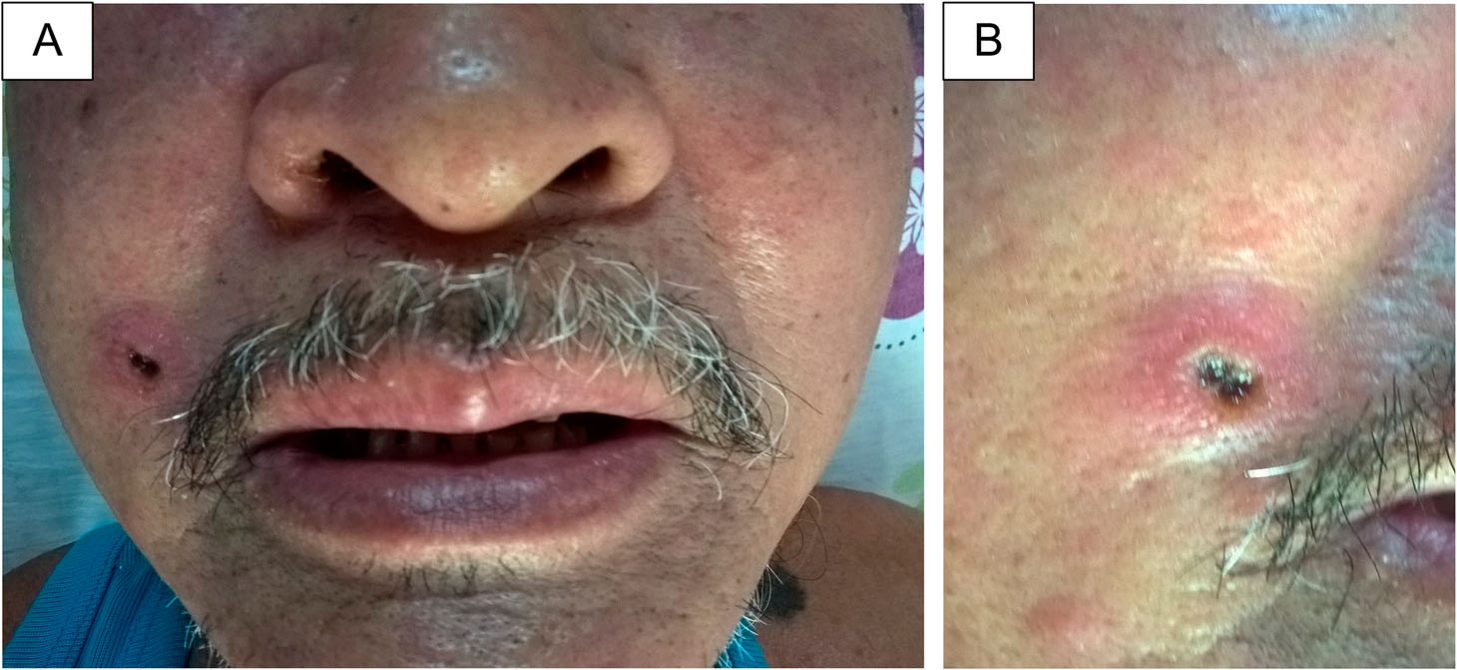
Eschar on the right face of a patient with *Rickettsia felis* infection in central Vietnam. Eschar on the right face (a), close-up view of the eschar (b).

*R. felis* was detected in two patients, who presented with continuous mild fever, a peak body temperature of 38°C–38.5°C, headache, and normal WBC, PLT, AST and ALT levels. One individual had an eschar with a 5 × 8-mm, painless, centred black crust surrounded by a red halo on the right side of his face (Figure 3). He also had a rash on his face and certain small swollen lymph nodes on his right neck. The remaining patient did not present with eschar, rash or lymphadenopathy.

### Antimicrobial treatment regimens

At least one empiric antibiotic was administered to 188 (49.7%) patients for a course of 5–10 days. Among these patients, most (152/188; 80.9%) received one antibiotic, and the others (36/188; 19.2%) received a combination of two or three antibiotics. Among the monotherapy cases, amoxicillin was the most commonly used antibiotic (43/152; 28.3%), followed by doxycycline (39/152; 25.7%), 2nd/3rd-generation cephalosporin (28/152; 18.4%), fluoroquinolones (22/152; 14.5%), macrolides (18/152; 11.8%), and antimalarial drugs (2/152; 1.3%). The most common combination antibiotic therapy was 2nd/3rd-generation cephalosporin and a fluoroquinolone (16/36; 44.4%), followed by 2nd/3rd-generation cephalosporin and doxycycline (6/36, 16.7%).

Twenty-nine of 41 (70.7%) patients with a rickettsial infection (16/21 cases of scrub typhus, 12/18 cases of murine typhus, and 1/2 cases of spotted fever caused by *R. felis*) received anti-rickettsial antibiotics (doxycycline [*n* = 23], azithromycin [*n* = 5], doxycycline and chloramphenicol [*n* = 1]). With the exception of one case of codetection of scrub typhus and leptospirosis, only six of the remaining 17 (35.3%) patients with leptospirosis received the appropriate antibiotics (doxycycline [*n* = 3], amoxicillin [*n* = 2], ceftizoxime [*n* = 1]). All the patients with influenza were not given anti-influenza agents, but approximately half of them (37/79; 46.8%) received an antibacterial agent (mainly 2nd/3rd-generation cephalosporin or amoxicillin). None of the patients died, and all the patients who either received an appropriate or inappropriate antibiotic had recovered completely from their illnesses at the time of discharge.

## Discussion

Attributing a detected pathogen to clinical diagnosis or to the cause of AUF is a major issue. Compared with the few similar studies in the literature, the inclusion of local afebrile controls in our study was essential for our interpretation and discussion of the results [8]. Our study allowed a final causative diagnosis to be made in over half (216/378; 57.1%) of the patients. A total of 185 patients were diagnosed with causes, and 31 patients were diagnosed with probable causes (Table 5). Indeed, rickettsial agents, *Leptospira*, dengue virus, influenza virus, adenovirus and enterovirus were considered causes of AUF because these pathogens are known as infectious agents of acute febrile illness, and their frequencies were significantly greater in AUFs than in Controls in this study. It was more difficult to conclude whether other respiratory pathogens that were present in similar proportions in AUFs and Controls could be causes of AUF. Thus, in AUF patients with these pathogens, the pathogen was considered a probable cause if the patient presented with cough, breathlessness or sore throat. During the study period, the clinicians did not make a causative diagnosis for these cases due to the lack of diagnostic tools and because the patients presented with sporadic symptoms that did not link to physical examination results associated with a syndrome.

**Table 5.** Causes and probable causes of AUF.

Diagnosis	*n*	%
One-pathogen detection (*n *= 144)		
*Causes*		
Rickettsial infections	40	10.6
*Orientia tsutsugamushi*	20	
*Rickettsia typhi*	18	
*Rickettsia felis*	2	
Dengue	28	7.4
*Leptospira*	12	3.2
Influenza	33	8.7
Influenza A	26	
Influenza B	7	
Adenovirus	9	2.4
Enterovirus	5	1.3
*Probable causes*		
Respiratory syncytial viruses	1	0.3
*Haemophilus influenzae* non-type B	2	0.5
*Klebsiella pneumoniae*	11	2.9
*Moraxella catarrhalis*	2	0.5
*Streptococcus pneumoniae*	1	0.3
Two-pathogen codetection (*n *= 51)		
*Causes*		
*Orientia tsutsugamushi *+ *Leptospira*	2	0.5
*Leptospira *+ RVI	4	1.1
*Leptospira* + Influenza A	1	
*Leptospira* + Influenza B	2	
*Leptospira* + Adenovirus	1	
Dengue + Adenovirus	1	0.3
Influenza + RBI	27	7.1
Influenza A + *Haemophilus influenzae* non-type B	5	
Influenza B + *Haemophilus influenzae* non-type B	1	
Influenza A + *Streptococcus pneumoniae*	4	
Influenza B + *Streptococcus pneumoniae*	1	
Influenza A + *Klebsiella pneumoniae*	12	
Influenza B + *Klebsiella pneumoniae*	3	
Influenza A + *Staphylococcus aureus*	1	
Other RVI + RBI	4	1.1
Enterovirus + *Haemophilus influenzae* type B	1	
Enterovirus + *Haemophilus influenzae* non-type B	1	
Adenovirus + *Streptococcus pneumoniae*	2	
*Probable causes*		
Human coronavirus 229E + *Streptococcus pneumoniae*	1	0.3
2 RBIs	12	3.2
*Streptococcus pneumoniae* *+* *Haemophilus influenzae* non-type B	3	
*Streptococcus pneumoniae* *+* *Klebsiella pneumoniae*	1	
*Klebsiella pneumoniae* *+* *Haemophilus influenzae* non-type B	5	
*Klebsiella pneumoniae* *+* *Mycoplasma pneumoniae*	1	
*Mycoplasma pneumoniae* *+* *Haemophilus influenzae* non-type B	1	
*Staphylococcus aureus* *+* *Haemophilus influenzae* non-type B	1	
Three-pathogen codetection (*n* = 22)		
*Causes*		
Influenza A + RPIs	16	4.2
Influenza A + Human coronavirus OC43 + *Klebsiella pneumoniae*	1	
Influenza A + *Staphylococcus aureus* *+* *Haemophilus influenzae* non-type B	1	
Influenza A + *Staphylococcus aureus* *+* *Klebsiella pneumoniae*	1	
Influenza A + *Haemophilus influenzae non-type B* + *Klebsiella pneumoniae*	6	
Influenza A + *Haemophilus influenzae non-type B* + *Streptococcus pneumoniae*	5	
Influenza A + *Streptococcus pneumoniae* + *Klebsiella pneumoniae*	2	
Adenovirus + RPIs	5	1.3
Adenovirus + Enterovirus + *Haemophilus influenzae* non-type B	1	
Adenovirus + *Streptococcus pneumoniae* + *Haemophilus influenzae* non-type B	2	
Adenovirus + *Klebsiella pneumoniae* + *Haemophilus influenzae* type B	1	
Adenovirus + *Streptococcus pneumoniae* + *Klebsiella pneumoniae*	1	
*Probable causes*		
*Mycoplasma pneumoniae* + *Streptococcus pneumoniae* + *Klebsiella pneumoniae*	1	0.3

Note: RVI: respiratory viral infection, RBI: respiratory bacterial infection, RPIs: respiratory pathogen infections.

Vector-borne infections, including emerging or re-emerging infections, were frequently identified as the cause of AUF, as previously reported in Asia [1,9].

### Rickettsial infections

Our study identified rickettsial agents in 10.8% of AUFs. Rickettsial infections are caused by obligate intracellular bacteria of the order Rickettsiales and are important causes of illness and death worldwide [3,10]. In Laos, a country that shares a border with Vietnam, rickettsial infections are known to be responsible for more than one-quarter (27%) of febrile adults with negative blood cultures [11]. Rickettsial infections are also emerging diseases among ill travellers returning from sub-Saharan Africa and Southeast Asia [12].

In Vietnam, scrub typhus caused by *O. tsutsugamushi* was identified as an important cause of acute illness in the US forces deployed to the country during the Vietnam War [13]. This disease is transmitted by the bite of trombiculid mites, commonly called chiggers, and may be fatal. The disease had been poorly studied in Vietnam until an imported case was reported in 1997 [14]. The recent, serological, hospital-based study of patients with clinically suspected rickettsioses in northern Vietnam conducted by Hamaguchi et al. revealed that 40.9% and 33.3% of patients had scrub typhus and murine typhus, respectively [15].

Murine typhus, a flea-borne rickettsiosis, was also recognized in the 1960s in Vietnam as a cause of undifferentiated febrile illness but remains poorly studied and diagnosed [16]. The ratio of scrub typhus to murine typhus obtained in the study conducted by Hamaguchi et al. was similar to that found in our study [15]. Therefore, the prevalence of murine typhus could be approximately equal to that of scrub typhus in Vietnam. In addition, murine typhus has been found to be responsible for nearly 10% of undifferentiated fever cases in Laos [11].

Spotted fever group (SFG) rickettsial infections are not well known in Vietnam. A recent serosurvey found that 1.7% of the healthy population in northern Vietnam has antibodies against SFG rickettsiae (using *R. conorii* antigen, which is the agent of the tick-borne Mediterranean spotted fever) [17]. In our study, *R. felis* was the only SFG rickettsia detected. *R. felis* infection has been identified as an emerging rickettsiosis worldwide and an important cause of AUF in sub-Saharan Africa [18,19]. Fleas have been recognized as vectors of *R. felis*, but mosquitoes, such as *Anopheles gambiae*, the major vector of malaria, may also transmit this agent [19]. Few cases of this emerging infection have been identified in Asia [18], and our three *R. felis* cases might be the first evidence of its existence in Vietnam. Because *R. felis* has also been detected in control afebrile patients in Africa [20] and in Asia, as in our study, further investigation is needed to precisely determine the role of *R. felis* in AUF in the tropics.

Q fever caused by *C. burnetii* occurs worldwide, is most frequently acquired through the inhalation of contaminated aerosols or the consumption of milk, and has been identified as an important cause of AUF [21–23]. Although it has been reported in some countries surrounding Vietnam [24], all our patients were negative for *C. burnetii*; therefore, we still do not have evidence of Q fever in Vietnam. Thus, this disease might exist with a very low prevalence, and further surveillance should be performed in the future.

Diagnosis of rickettsial infections may be difficult in clinical practice due to the diversity of manifestations, particularly in developing countries where diagnostic tests are usually lacking. Typical clinical signs, including eschar and skin rash, were rarely observed in a patient with dual genotype of *O. tsutsugamushi* infection [25]. In most cases, typical clinical signs of rickettsial infections might be absent or missed, resulting in the misdiagnosis of patients as having AUF. A finding of skin rash or eschar can increase the accuracy of the diagnosis of rickettsial infections. Here, among 16 AUF patients with rash, four patients had rickettsial infections (three cases of murine typhus and one case of scrub typhus), one had leptospirosis, one was coinfected with scrub typhus and leptospirosis, and 10 patients had an undetected cause. The appearance of rash in scrub typhus varies depending on the *O. tsutsugamushi* genotype and might be less frequent in Vietnamese patients with scrub typhus [25,26]. Among the patients with scrub typhus, eschar was present in 47.4% of patients, similar to the proportion observed in our previous study [26].

One patient with *R. felis* infection presented with an eschar that generally resembled one found in cases of scrub typhus. Because the local clinicians are unaware of the existence of *R. felis* but are aware of scrub typhus, the patient was diagnosed with suspected scrub typhus and immediately prescribed doxycycline. An eschar sometimes appears in *R. felis* infection [27] and is an important diagnostic sign. Skin rash might present as maculopapular in 70% of patients with *R. felis* infection [18,28], but this symptom might also be absent. However, it appears that studies reporting the absence of rash included limited cases of *R. felis* infections [29,30]. We believe that the suggestive manifestations (e.g. eschar and rash) of *R. felis* infection might be observed in a significant proportion of patients, but these are insufficiently reported due to the lack of a careful examination and the absence of epidemiological information. Because *R. felis* is an emerging agent and is known to be a cause of AUF in Vietnam, its presence should be examined in such patients with AUF, including local patients and travellers who returned from Vietnam and similar areas.

Moreover, among the 12 patients presenting with an eschar in our study, 10 rickettsial infection cases were confirmed (nine cases of *O. tsutsugamushi* and one case of *R. felis* infection), and two remaining cases were clinically suspected rickettsial infections. All 12 patients were completely recovered after a doxycycline course. In fact, the diagnostic tools for detecting infectious aetiologies are insufficient in almost all hospitals in Vietnam and other developing countries; therefore, clinicians treating a patient with fever and eschar should not delay prescribing doxycycline against rickettsial agents. When diagnosing a patient with AUF, a careful examination focusing on the occurrence of eschar, rash, and abnormal aminotransferase levels might increase the precision of rickettsial infection diagnosis.

### Leptospirosis

Leptospirosis is one of the most common zoonotic diseases worldwide, and this potentially fatal disease is considered a public health issue in Asia [3,9]. This infection is also known as an important cause of fever in returning travellers [31]. Despite its high seroprevalence in humans and animals in Vietnam, very few cases have been documented [32]. We detected *Leptospira* DNA in 4.8% of AUFs and 0.8% of Controls. During this study, the clinicians never suspected any cases of leptospirosis among the 18 cases with confirmed leptospirosis, but they suspected that three of these cases were scrub typhus and prescribed doxycycline. These patients presented a medium febrile course, and two-thirds of these patients had a headache. Other basic laboratory results, such as the WBC count, PLT count and serum aminotransaminase levels, were normal, which did not aid the diagnosis of the disease. Thus, clinicians may, in such cases, diagnose leptospirosis, but they must nevertheless pay particular attention to the illness process in these patients and the exclusion of other causes.

### Dengue fever and absence of other arboviral infections

Dengue infection remains a major health problem in Vietnam, where its incidence has increased over the past three decades (annual average percentage change from 1980 to 2010: 10.4%) [33]. Dengue cases occur throughout the year, but peaks are detected between June and October [34]. The dengue proportion of 11.1% detected in our study is underestimated because patients with dengue-positive RDTs were excluded from the study. The known sensitivity (6.4–81.5%) of the RDTs could lead to the misdiagnosis of dengue infection cases [35,36]. A clinical analysis focusing on 28 patients with dengue monoinfection showed almost nonspecific manifestations, which were difficult to distinguish from other causes, even in two patients with petechiae (subcutaneous haemorrhages). Although thrombopenia and leukopoenia are not specific for dengue infection, these manifestations were observed more frequently in dengue cases than in the other cases included in our study.

Chikungunya virus was not identified in our study. This virus is reported to be in frequent circulation and to have caused certain major outbreaks in Southeast Asia [37,38]. It is also described as a cause of fevers of unknown origin in American soldiers in Vietnam during the Vietnam War [39]. This disease and dengue fever are both transmitted by *Aedes* mosquitoes and have similar symptoms, which makes it easy to confuse these diseases and underrecognize chikungunya cases in dengue hyperendemic areas [40]. However, the most recent systematic review found no evidence of recently sustained transmission of chikungunya in central and southern Vietnam [41].

Zika virus, another arbovirus transmitted by *Aedes* and other mosquitoes (e.g. *Mansonia uniformis*, *Culex perfuscus*, *Anopheles coustani*) [42,43], was also not detected in our study. This virus has recently become one of the most widely spread arboviruses throughout the world [43]. Since 2015, some tourists have been found to be infected with Zika virus while travelling in Vietnam [44–47], and hundreds of local cases have been reported in the South and Central Highlands regions since 2016; however, no case has been reported in the north of the country prior to this study [48].

### Influenza

Similar to some previous studies in Southeast Asia, our study illustrates that influenza is a major cause (20.9%) of AUF [7,49,50]. None of the 384 throat swabs from Controls were positive for influenza, even though afebrile patients may carry the virus [7]. Although influenza is reputedly linked to cold weather in temperate regions [51], it occurs throughout the year in tropical regions such as Vietnam [50]. Because of the high proportion of coinfection with influenza virus and bacteria observed in this study (Table 5), antibiotics might be considered in several cases, particularly in elderly or risk-associated patients, because the majority of influenza-related deaths are caused by bacterial superinfection [52]. An influenza vaccination programme should be discussed for the local population in Vietnam, at least for the populations at high risk for severe influenza, including pregnant women, children aged <5 years, the elderly, and individuals with underlying health conditions [53].

### Other respiratory infections

Adenovirus and enterovirus were also important causes of AUF (6.9% in AUFs vs. 1% in Controls). These respiratory viruses and influenza viruses are responsible for the majority of acute illnesses at all ages worldwide [54]. Several cases of viruses (RSV [*n *= 1] and coronavirus [*n *= 2]) were considered probable causes in this study because they were detected in patients who presented with cough, breathlessness or sore throat. RSV is the most common cause of acute respiratory infection in young children [55], but it does not appear to play an important role in adults with AUF, as confirmed in this study. Similarly, other viruses (parainfluenza virus, rhinovirus, coronavirus) were also less frequently detected in both groups in our study. However, respiratory viruses usually exhibit seasonal variations [54], and thus, a few months of sampling might not yield sufficient information regarding the prevalence of these viruses.

The bacteria in throat swabs were detected at similarly high proportions in AUFs and Controls. Some cases of bacteria (*K. pneumoniae* [*n* = 46], *H. influenzae* [*n* = 36], *S. pneumoniae* [*n* = 24], *S. aureus* [*n* = 4], *M.* pneumoniae [*n* = 3], and *M. catarrhalis* [*n* = 2]) detected in AUF patients presenting with accompanying respiratory symptoms were considered probable causes of AUF. With the exception of these probable cases, most of the detected bacteria might be considered colonizing bacteria. However, we noticed that *K. pneumoniae*, which is known to be associated with pneumonia, urinary tract infection and, particularly in Asia, pyogenic liver abscesses [56], was detected in more than one-quarter of all the participants (209/762, 27%) in this study. Thus, further studies of this bacterium might be useful to determine its effects on liver abscess conditions in the population in this area.

### Codetections or coinfections?

The codetection of *O. tsutsugamushi* and *Leptospira* in two patients could be considered a coinfection, as documented previously [7]. Almost all other cases of codetections of respiratory viruses and bacteria might also be coinfections. Unlike other studies that used serological tests, which might yield false-positive results due to cross-reacting antibodies or the nonspecific polyclonal immunoreactivity of different aetiologies [57], we used a qPCR-direct method for pathogen detection and thus obtained strong evidence of the presence of the detected pathogens in the codetection cases. However, the results should be carefully interpreted because the presence of a microorganism in a febrile patient does not always indicate that it is a cause of the fever, and PCR cannot differentiate between infection and carriage or colonization. Thus, we first assessed every detected virus or bacterium in comparison to its frequency in AUFs and Controls, and we then evaluated the combination of positive clinical manifestations in the febrile patients and the presence of viruses or bacteria if their presence was not significantly different between the AUFs and Controls. Accordingly, although the detected frequency of various viruses and bacteria, particularly in throat swabs, was significant, we considered only 73 codetections, which consisted of coinfection with two or three pathogens, as causes or probable causes of AUF (Table 5).

### Factors that might be diagnosis predictors of common causes of AUF

In the effort to identify clinical predictors of some common causes of AUF that could help clinicians differentiate AUF, we performed multivariate analyses for each cause consisting of infection with a single pathogen (Table S3). Accordingly, eschar and a high ALT level (>80 IU/L) were associated with scrub typhus, whereas a high AST level (>80 IU/L), a long fever duration (>7 days) and a job as a farmer were associated with non-scrub typhus rickettsial infections. Only splenomegaly was associated with leptospirosis. Haemorrhage, low WBC count (<4 k/µL) and low PLT count (<150 k/µL) were associated with dengue infection. The presence of cough was associated with influenza, whereas a young age (<30 years) was associated with noninfluenza respiratory viral infection. Although a limited number of cases were included in each logistic regression model, we believe that these results might be useful for local clinicians who care daily for patients with AUF but have limited diagnostic tools to differentiate some common causes of AUF from other causes.

### Study limitations

Even though we were able to detect aetiologies in a large number of AUFs, this study has some limitations. The sampling period was limited to 4 months, which does not provide information on the seasonal circulation of aetiologies. The serological test was not applied for all pathogens except rickettsial agents, which might have led to missed diagnoses of some infections. Finally, blood culture was not performed regularly due to the limited facilities during the sampling in local laboratories. Thus, we might also have missed some typhoid cases and some other bacteria, as they were found in 1–8% in studies in which blood culture was used routinely [58,59].

## Conclusion

Epidemiological studies on fever aetiologies in the rural tropics are highly needed. The extensive and systematic workup of this study provided unique data on the causes of AUF in Vietnam because we used reference methods and local afebrile subjects as controls, which allowed us to better clarify differences between infection and colonization. These data have implications for the management of patients with AUF in Vietnam and even Southeast Asia. We recommend that in addition to RDTs for dengue and microscopy for malaria, local hospitals should be equipped with diagnostic tools (e.g. RDTs, serological tests, and molecular biology tests) for rickettsial infections, leptospirosis, and influenza. More importantly, a significant proportion (15.6% if we include rickettsial diseases and leptospirosis) of the causative agents of AUF are susceptible to doxycycline. Therefore, this drug needs to be included in the panel used to treat patients with AUF. Empirical treatment with a combination of β-lactam (e.g. ceftriaxone) and doxycycline might be used to treat AUF patients with a severe clinical presentation because some agents detected here could result in fatality.

## Materials and methods

### Study design and population

This prospective case-control study was conducted from May to August 2016 at four major general hospitals in Quang Nam province – a rural area located in central Vietnam (Figure S2). These included Quang Nam Central General Hospital, Quang Nam Provincial General Hospital, Quang Nam Northern Mountainous Region General Hospital and Quang Nam Regional General Hospital, which have approximately 2130 beds combined and serve 1,487,700 residents of Quang Nam province and thousands of people in the neighbouring districts of Quang Ngai province.

### Participant enrolment

All patients who met the eligibility criteria during the study period were continuously enrolled. AUFs were included in this study if they fulfilled four primary criteria: (i) age ≥15 years; (ii) axillary temperature ≥38°C; (iii) duration of fever ≤21 days [60]; and (iv) no evidence of localized infection based on history, initial physical examination, complete blood count, chemistry profile, urinalysis or chest radiography. Some patients who presented with several sporadic symptoms (e.g. cough, sore throat, breathlessness, diarrhea, and abdominal pain) that were not consistent with physical examination results in a clinical diagnosis were also included in the study. We excluded dengue fever and malaria because these diseases are well known to be common causes of febrile illness in Vietnam, and their diagnostic tests are available in almost all hospitals in Vietnam. Thus, we excluded patients who tested positive for malaria on a peripheral blood smear (Giemsa stained thick and thin films) or for dengue on RDTs for nonstructural 1 glycoprotein (NS1) antigen/IgM. We also excluded patients with other conditions that could bias the results, such as receiving cancer chemotherapy or immunosuppressive therapy, having HIV infection, and hospitalized for ≥72 h within the preceding 30 days. HIV testing was not performed routinely. We performed HIV ELISA testing only for patients who had never been tested for HIV infection if their clinical symptoms and signs suggested an immunosuppression condition. To ensure a complete follow-up during the illness course, only inpatients with AUF admitted to infectious disease wards were considered for this study.

In parallel, the controls were ≥15-year-old afebrile patients who visited due to noninfectious medical problems, such as cardiovascular diseases, trauma, ophthalmological disorders, etc. We excluded individuals accompanying sick family members and those who had a history of fever within the past seven days to minimize the possibility of infectious diseases in the incubation period. The Controls were either outpatients or inpatients in various wards other than the infectious disease ward.

### Clinical data and sample collection

All clinicians and nurses who participated in this study were trained on the study protocol and sample collection. A clinician examined the patients and invited them to participate in the study if they met the eligibility criteria. In particular for the AUFs, all the patients were screened for dengue infection by RDTs, and the patients who came from a malaria endemic area were screened using a peripheral blood smear. Accordingly, only AUF patients who were negative for these diseases were included in this study. After obtaining written informed consent from the participants, the clinicians recorded their vital signs, signs and symptoms of the current illness, their history of exposure to the forest within 30 days, chronic comorbidity diseases and basic clinical laboratory results on a case report form (CRF). A nurse collected a total of 3 mL of whole blood, divided into 1 mL of whole blood in an EDTA tube and approximately 1 mL of plasma in a dry tube after centrifugation, and 1 mL of urine from each participant. One throat swab specimen was taken by a clinician and preserved immediately into a viral transport medium. The clinician also collected eschar swab specimens if the patient presented with one or several eschars based on a previously described method [26]. In addition, from each patient with AUF, we collected 1 mL of plasma in the convalescent phase, which occurred 7–10 days after the first plasma collection. All the specimens were immediately preserved at −20°C at the hospitals in Quang Nam province (Vietnam) until analysis at the University Hospital Institute *Méditerranée Infection*, Marseille (France).

### Molecular biology analyses

For each patient, 200 µL of whole blood and 200 µL of urine were used for DNA extraction, and 200 µL of acute-phase plasma and 200 µL of the throat swab sample in viral transport media were used for DNA/RNA extraction. The eschar specimens were pretreated and used for DNA extraction as previously described [26]. DNA extraction using the DNA EZ1 extraction kit and DNA/RNA extraction using the EZ1 Virus Mini Kit v2.0 (Qiagen, Hilden, Germany) were performed with the EZ1 Advanced XL Robot (Qiagen) according to the manufacturer’s instructions. The DNA and DNA/RNA products were either immediately used or stored at −20°C and at −80°C until molecular analysis, respectively. To avoid cross-contamination, the nucleic acid-extracting EZI Advanced XL Robot was disinfected after each batch of extraction based on the manufacturer’s recommendations.

The DNA products isolated from the whole blood specimens were tested using quantitative real-time PCR (qPCR) with genus-specific primers and probes targeting specific sequences of *O. tsutsugamushi, Rickettsia* spp., *R. felis, R. typhi, Anaplasma* spp.*, Bartonella* spp., *Borrelia* spp., and *C. burnetii* (Table S4). These DNA products were also tested by qPCR for *Leptospira* spp., *Salmonella* spp., *Salmonella enterica* serovar Typhi/Paratyphi, *Shigella* spp., *S. aureus*, *S. pneumoniae*, *Streptococcus pyogenes*, *Tropheryma whipplei* and *Burkholderia pseudomallei*. The DNA products isolated from the eschar swab specimens were tested for *O. tsutsugamushi, Rickettsia* spp., *R. felis* and *R. typhi,* whereas the DNA products isolated from urine specimens were tested for *Leptospira* spp. using specific qPCR systems. The DNA/RNA products obtained from the plasma samples were tested by real-time reverse-transcription PCR (qRT-PCR) for dengue viruses (Dengue-1 virus, Dengue-2 virus, Dengue-3 virus and Dengue-4 virus), chikungunya virus, Zika virus and hantaviruses (Dobrava virus, Puumala virus, Tula virus and Hantaan/Seoul virus). The Eurogentec Takyon qPCR kit (Eurogentec, Belgium) was used for bacterial DNA detection, and the LightCycler^®^ Multiplex RNA Virus Master (Roche Diagnostic, Germany) was used for viral RNA detection.

The Multiplex TaqMan real-time PCR using the FTD^®^ respiratory pathogens-33 kit (Fast-track Diagnostics, Luxembourg) was used to detect various respiratory viruses, bacteria and fungi in the DNA/RNA products obtained from throat swabs. The targeted pathogens were influenza A virus; influenza B virus; influenza C virus; influenza A (H1N1) swl virus; human parainfluenza viruses 1, 2, 3 and 4; human coronaviruses NL63, 229E, OC43 and HKU1; human metapneumoviruses A/B; human rhinovirus; human RSV A/B (RSV A/B); human adenovirus; enterovirus; human parechovirus; human bocavirus; *M. pneumoniae*; *Chlamydia pneumoniae*; *S. pneumoniae*; *H. influenzae*; *H. influenzae* type B; *S. aureus*; *M. catarrhalis*; *Bordetella* spp.; *K. pneumoniae*; *Legionella pneumophila/longbeachae*; *Salmonella* spp.; and *Pneumocystis jirovecii.* PCR assays were performed with a CFX Connect^TM^ Real-Time PCR Detection System (Bio-Rad, USA). Negative control and positive controls for the corresponding pathogens were included in each run. The PCR procedures were performed according to the manufacturer’s instructions. Quantitative PCR and quantitative reverse-transcription PCR assays were considered positive if the cycle threshold (Ct-value) was <35. All the AUFs and Controls were tested in the same panel of these pathogens.

### Serology analyses

The acute- and convalescent-phase plasma samples from all the patients with AUF were subjected to specific indirect immunofluorescence (IFA) assays to detect antibodies of *Orientia tsutsugamushi* serotypes Karp, Kato, and Gilliam; *Rickettsia typhi*; *Rickettsia felis*, *Rickettsia conorii* and another SFG rickettsiae; and *Coxiella burnetii* as previously described [26]. An IFA result was considered positive (acute infection) in any of the following cases: (i) detection of IgM, (ii) seroconversion between acute and convalescent sera, or (iii) 2-fold increased IgG and/or IgM titres between acute- and convalescent-phase sera. The cut-offs were those that had been validated and were used in our reference centre for the diagnosis of rickettsial diseases as follows: IgM titre >1:64 for *R. conorii*; IgM titre >1:32 for other rickettsial antigens; IgG titre >1:128; IgG titres >1:64 for other rickettsial antigens [61,62]. Western immunoblotting was used to detect *Rickettsia* spp. in cases that were not differentiated by IFA due to cross-reactivity of relevant antigens of *Rickettsia* spp. [11,61].

### Statistical analysis

The data were entered and verified using Microsoft Excel software and analysed using Stata version 12.0 (StataCorp, Texas 77845, USA) and R version 3.1.3 (R Foundation for Statistical Computing, Vienna, Austria). The categorical variables are summarized as frequencies and percentages. Different groups were compared using the *χ*^2^ test and Fisher’s exact test. Student’s *t*-test, the Wilcoxon–Mann–Whitney test and the Kruskal–Wallis test were used to compare different continuous variable between the groups. A *p*-value of less than .05 was considered statistically significant.

### Ethics agreement

This study was approved by the Ethical Review Committee of the Quang Nam Central General Hospital of the Ministry of Health, Vietnam (approval no. 01/HDDD-2016). All the patients included in this study provided written informed consent.

## Supplementary Material

Supplemental Material
