# Supplementary material for: Prospective case-control analysis of the aetiologies of acute undifferentiated fever in Vietnam
Source: Emerg Microbes Infect. 2019 Mar 4;8(1):339–52. doi: 10.1080/22221751.2019.1580539 (PMC6455186; doi:10.1080/22221751.2019.1580539)
Supplement: Supplemental Material [file TEMI_A_1580539_SM7335.zip › Supplementary files/Table S2. Correlation between proportions of viral and bacterial infections in AUF.docx]

**Table S2. Correlation between frequencies of the bacteria and viruses detected in throat swabs of patients with AUF**

|  | All bacteria | | ***M. pneumoniae*** | | ***S.aureus*** | | ***H. influenzae*** | | ***S. pneumoniae*** | | ***K. pneumoniae*** | | ***M. catarrhalis*** | |
| --- | --- | --- | --- | --- | --- | --- | --- | --- | --- | --- | --- | --- | --- | --- |
|  | Cor index | p | Cor index | P | Cor index | P | Cor index | p | Cor index | p | Cor index | p | Cor index | p |
| All viruses | 0.018 | 0.73 | -0.057 | 0.26 | -0.034 | 0.51 | 0.070 | 0.17 | 0.076 | 0.14 | -0.022 | 0.67 | -0.081 | 0.11 |
| Influenza A&B | 0.071 | 0.17 | -0.046 | 0.37 | 0.007 | 0.89 | 0.045 | 0.38 | 0.049 | 0.34 | 0.046 | 0.37 | -0.065 | 0.21 |
| Influenza A | 0.079 | 0.12 | -0.041 | 0.43 | 0.030 | 0.56 | 0.079 | 0.13 | 0.050 | 0.34 | 0.050 | 0.33 | -0.058 | 0.26 |
| Influenza B | -0.005 | 0.92 | -0.018 | 0.73 | -0.044 | 0.40 | -0.060 | 0.24 | 0.006 | 0.91 | -0.001 | 0.98 | -0.025 | 0.63 |
| Parainfluenza 1^a^ | 0.050 | 0.33 | -0.005 | 0.93 | -0.012 | 0.82 | **0.105** | **0.04** | **0.132** | **0.01** | 0.081 | 0.12 | -0.007 | 0.90 |
| Enterovirus | -0.041 | 0.43 | -0.013 | 0.80 | -0.033 | 0.52 | 0.068 | 0.19 | -0.057 | 0.27 | -0.094 | 0.07 | -0.019 | 0.72 |
| Adenovirus | -0.056 | 0.28 | -0.020 | 0.70 | -0.050 | 0.33 | 0.016 | 0.75 | 0.096 | 0.06 | -0.087 | 0.09 | -0.028 | 0.58 |
| RSV A/B | -0.075 | 0.14 | -0.007 | 0.90 | -0.016 | 0.75 | -0.036 | 0.49 | -0.028 | 0.58 | -0.046 | 0.37 | -0.009 | 0.86 |
| Coronavirus | 0.049 | 0.34 | -0.009 | 0.86 | -0.023 | 0.65 | 0.015 | 0.77 | -0.040 | 0.43 | 0.048 | 0.35 | -0.013 | 0.80 |
| ^a^Only one case of Parainfluenza 1 was detected that could not validate the association between the virus and bacterium | | | | | | | | | | | | | | |
