# Supplementary material for: Prospective case-control analysis of the aetiologies of acute undifferentiated fever in Vietnam
Source: Emerg Microbes Infect. 2019 Mar 4;8(1):339–52. doi: 10.1080/22221751.2019.1580539 (PMC6455186; doi:10.1080/22221751.2019.1580539)
Supplement: Supplemental Material [file TEMI_A_1580539_SM7335.zip › Supplementary files/Table S3. Clinical predictors of some common causes of AUF.docx]

**Table S3. Clinical predictors of common causes of AUF ^a^**

| **ST (n=19) vs Other AUF (n=359)** | **Adjusted Odds Ratio (OR)** | **95% Confidence Interval (CI)** | **p-value** |
| --- | --- | --- | --- |
| Rigor | 4.7 | 0.9-36.1 | 0.09 |
| Eschar | **71.3** | **5.9-2006.7** | **0.002** |
| Nausea/vomiting | 0 | ̶ | 0.9 |
| ALT>80 UI/L | **23.2** | **4.8-172.0** | **<0.001** |
| **Non-ST RI (n=20) vs Other AUF (n=358)** |  |  |  |
| Farmer | **10.2** | **2.0-94.4** | **0.02** |
| Forest exposure | 0 | ̶ | 0.9 |
| Fever duration>7 days | **8.9** | **2.2-41.1** | **0.003** |
| AST>80 UI/L | **12.7** | **2.7-66.9** | **0.001** |
| **Lepto (n=12) vs Other AUF (n=366)** |  |  |  |
| Farmer | 4.4 | 0.9-39.1 | 0.1 |
| Fever duration>7 days | 0 | ̶ | 0.9 |
| Splenomegaly | **59.7** | **1.7-2378.3** | **0.01** |
| Hepatomegaly | 0 | ̶ | 0.9 |
| WBC<4 k/µL | 0 | ̶ | 0.9 |
| Lymphocyte<1 k/µL | 4.6 | 0.9-32.8 | 0.08 |
| ALT>80 UI/L | 0 | ̶ | 0.9 |
| **DF (n=28) vs Other AUF (n=350)** |  |  |  |
| Continuous fever | 2.2 | 0.8-7.2 | 0.2 |
| Hemorrhage | **16.4** | **1.6-136.0** | **0.01** |
| WBC<4 k/µL | **3.5** | **1.1-10.8** | **0.03** |
| PLT<150 k/µL | **5.7** | **1.8-22.1** | **0.005** |
| **Flu (n=33) vs Other AUF (n=345)** |  |  |  |
| Dizziness | 2.6 | 0.9-8.0 | 0.1 |
| Disappetite | 0.4 | 0.1-1.3 | 0.1 |
| Cough | **5.2** | **1.8-16.2** | **0.003** |
| Abdominal pain | 2.7 | 0.7-9.0 | 0.1 |
| WBC<4 k/µL | 4.9 | 1.2-17.7 | 0.06 |
| ALT>80 UI/L | 0 | ̶ | 0.9 |
| **Nonflu RVI (n=14) vs Other AUF (n=364)** |  |  |  |
| Age<30 (years) | **10.0** | **2.6-65.2** | **0.003** |
| Lymphocyte<1 k/µL | 0.2 | 0-0.9 | 0.1 |
| PLT<150 k/µL | 0.3 | 0-1.2 | 0.1 |
| ST: scrub typhus, Non-ST RI: non-scrub typhus rickettsial infections, DF: dengue fever, Lepto: leptospirosis, Flu: influenza, Nonflu RVI: noninfluenza respiratory viral infection, Other AUF: other acute undifferentiated fever  ^a^Multivariate analyses for each of cause due to single pathogen infection were performed to investigate the clinical predictors of these infections in AUF. Given the high number of potential predictors for each diagnosis, a backward stepwise algorithm and the Akaike information criterion minimization [^1^](#_ENREF_1) were used to select the best regression models. Finally, odds ratios (ORs) and their 95% confidence intervals (CIs) were calculated. | | | |

**Supplementary reference**

1 Akaike, H. A new look at the statistical model identification. *IEEE Trans Autom Control* **19**, 716–723 (1974).
