# Supplementary material for: Prospective case-control analysis of the aetiologies of acute undifferentiated fever in Vietnam
Source: Emerg Microbes Infect. 2019 Mar 4;8(1):339–52. doi: 10.1080/22221751.2019.1580539 (PMC6455186; doi:10.1080/22221751.2019.1580539)
Supplement: Supplemental Material [file TEMI_A_1580539_SM7335.zip › Supplementary files/Table S4. Targeted pathogens and primers for singleplex qPCR.docx]

**Table S4. Targeted pathogens and primers for singleplex qPCR**

| **Targeted pathogen** | **Gene name** | **Primer system** | **Sequences** |
| --- | --- | --- | --- |
| *Orientia tsutsugamushi* | Periplasmic serine protease | Forward | AACTGATTTTATTCAAACTAATGCT |
|  |  | Reverse | TATGCCTGAGTAAGATACRTGAATRGAATT |
|  |  | Probe | 6FAM-TGGGTAGCTTTGGTGGACCGATGTTTAATCT |
| *Rickettsia felis* | Guanosine | Forward | GCATATACTTTATTGTGCGCAAGTT |
|  |  | Reverse | TTTATCGATTGACAGAAGAAGAAATCA |
|  |  | Probe | 6FAM-TCGCTTTTTGGGATTGTTTGCCAGA |
| *Rickettsia typhi* | Hypothetical protein | Forward | TGTCAGATTATAAAGACGATGCTCAGA |
|  |  | Reverse | GCAGCTTGTACTCCTTTAATTTGTTC |
|  |  | Probe | 6FAM-CCGCTACCGCAAATCCATCAGA |
| *Rickettsia* spp. | gltA | Forward | GTGAATGAAAGATTACACTATTTAT |
|  |  | Reverse | GTATCTTAGCAATCATTCTAATAGC |
|  |  | Probe | 6FAM-CTATTATGCTTGCGGCTGTCGGTTC |
| Anaplasmataceae | 23S | Forward | TGACAGCGTACCTTTTGCAT |
|  |  | Reverse | GTAACAGGTTCGGTCCTCCA |
|  |  | Probe | 6FAM-GGATTAGACCCGAAACCAAG |
| *Bartonella* spp. | ITS | Forward | GGGGCCGTAGCTCAGCTG |
|  |  | Reverse | TGAATATATCTTCTCTTCACAATTTC |
|  |  | Probe | 6FAM-CGATCCCGTCCGGCTCCACCA |
| *Borrelia* spp. | 16S | Forward | AGCCTTTAAAGCTTCGCTTGTAG |
|  |  | Reverse | GCCTCCCGTAGGAGTCTGG |
|  |  | Probe | 6FAM-CCGGCCTGAGAGGGTGAACGG |
| *Coxiella burnetii* | Hypothetical protein | Forward | CGCTGACCTACAGAAATATGTCC |
|  |  | Reverse | GGGGTAAGTAAATAATACCTTCTGG |
|  |  | Probe | 6FAM-CATGAAGCGATTTATCAATACGTGTATGC |
| *Staphylococcus aureus* | NucA_2 | Forward | GTTGTGGATGGTGATACATTTATTGC |
|  |  | Reverse | CCAAATGGTTGTACAGGCGTATTC |
|  |  | Probe | 6FAM-AGGCTTATAGGGGTTGATACGCCAGAAACGG |
| *Streptococcus pneumoniae* | lytA-CDC | Forward | ACGCAATCTAGCAGATGAAGCA |
|  |  | Reverse | TCGTGCGTTTTAATTCCAGCT |
|  |  | Probe | 6FAM-TGCCGAAAACGCTTGATACAGGGAG |
| *Streptococcus pyogenes* | Hypothetical protein | Forward | ACAGGAACTAATACTGATTGGAAAGG |
|  |  | Reverse | TGTAAAGTGAAAATAGCAGCTCTAGCA |
|  |  | Probe | 6FAM-AAAATGTTGTGTTTTAGGCACTGGCGG |
| *Tropheryma whipplei* | WiSP family protein | Forward | TGAGGATGTATCTGTGTATGGGACA |
|  |  | Reverse | TCCTGTTACAAGCAGTACAAAACAAA |
|  |  | Probe | 6FAM-GAGAGATGGGGTGCAGGACAGGG |
| *Salmonella* spp. | invA | Forward | TCTGTTTACCGGGCATACCA |
|  |  | Reverse | CACCGTGGTCCAGTTTATCG |
|  |  | Probe | 6FAM-CCAGAGAAAATCGGGCCGCG |
| *Salmonella typhi* | Hypothetical protein | Forward | TCTCATGCTGCGACCTCAAA |
|  |  | Reverse | TTCATCCTGGTCCGGTGTCT |
|  |  | Probe | 6FAM-GCTTTTTGTGAAGCAACGCTGGCA |
| *Salmonella Typhi/Paratyphi* | narG | Forward | GCGCCACATCTTCATCAGAC |
|  |  | Reverse | CCCGTCCTGATATGCCAAAC |
|  |  | Probe | 6FAM-AGTAACTTGCCCCGCGCGGG |
| *Shigella* spp. | ipaH | Forward | CCTTTTCCGCGTTCCTTGA |
|  |  | Reverse | CGGAATCCGGAGGTATTGC |
|  |  | Probe | 6FAM-CGCCTTTCCGATACGTCTCTGCA |
| *Burkholderia pseudomallei* | Type III secretion system | Forward | CGTCTCTATACTGTCGAGCAATCG |
|  |  | Reverse | CGTGCACACCGGTCAGTATC |
|  |  | Probe | 6FAM-CCGGAATCTGGATCACCACCACTTTCC |
| *Leptospira* spp. | 16S | Forward | CCCGCGTCCGATTAG |
|  |  | Reverse | TCCATTGTGGCCGRACAC |
|  |  | Probe | 6FAM- CTCACCAAGGCGACGATCGGTAGC |
| Dengue-1 virus | Polyprotein gene | Forward | AGCATRAGGAGCATGGTCAC |
|  |  | Reverse | ATACCYCCAACAGCAGGAATT |
|  |  | Probe | 6FAM-TTGGCTAGATGGRGCTCATTCAAGAAGAAT |
| Dengue-2 virus | Polyprotein gene | Forward | CGYCCYTGCAGCATTCCAA |
|  |  | Reverse | TGGACCGACAAAGACAGATTCTT |
|  |  | Probe | 6FAM-CGCGAGAGAAACCGCGTGTCRACTGT |
| Dengue-3 virus | Polyprotein gene | Forward | TTGAGAATCTCTTCGCCAACTG |
|  |  | Reverse | AAGACGGGAAAACCGTCTATCAA |
|  |  | Probe | 6FAM-ATGCTGAAACGCGTGAGAAACCGTGT |
| Dengue-4 virus | Polyprotein gene | Forward | GGAGGGGTCTCCTCTAACCR |
|  |  | Reverse | TKGAGCAAACCGTGCTGCCT |
|  |  | Probe | 6FAM-GGGAGGCCATGCGCCACGGAAGC |
| Chikungunya virus | E1 gene | Forward | CCAAATTGTCCYGGTCTTCCT |
|  |  | Reverse | AAGCTYCGCGTCCTTTACCAAG |
|  |  | Probe | 6FAM-CCAATGTCYTCMGCCTGGACACCTTT |
| Zika virus | Polyprotein gene | Forward | CTTGGAGTGCTTGTGATT |
|  |  | Reverse | CTCCTCCAGTGTTCATTT |
|  |  | Probe | 6FAM-AAGAAGAGAATGACCACAAAGATCATC |
| Dobrava virus | DOBV | Forward | GACTCACCRTCATCAATYTGGGT |
|  |  | Reverse | GATGCCATGATIGTRTTCCTCAT |
|  |  | Probe | TCTGCCATGCCTGC |
| Puumala virus | PUUV | Forward | GARRTGGACCCRGATGACGTTAA |
|  |  | Reverse | CCKGGACACAYCATCTGCCAT |
|  |  | Probe | CAACARACAGTGTCAGCA |
| Tula virus | TULV | Forward | GCCGACCCRGATGATGTTAA |
|  |  | Reverse | CAAGYTGYCTCTTGAADTCTGCCA |
|  |  | Probe | TCCAATGCTGACACAGC |
| Hantaan/Seoul virus | HNTV/SEO | Forward | CATGGCWTCHAAAGACWGTGGG |
|  |  | Reverse | TTKCCCCAGGCAACCAT |
|  |  | Probe | TCAATGGGRATACAACT |
